# Free Intra-Abdominal Air without Peritoneal Perforation after TEM: A Report of Two Cases

**DOI:** 10.1155/2012/185429

**Published:** 2012-12-17

**Authors:** Rutger J. Franken, Daan E. Moes, Yair I. Z. Acherman, Eric J. Derksen

**Affiliations:** Department of Surgery, Slotervaart Hospital, Louwesweg 6, 1066 EC Amsterdam, The Netherlands

## Abstract

Transanal endoscopic microsurgery (TEM) is a minimally invasive treatment modality for a variety of rectal lesions. Due to its minimally invasive nature, TEM has emerged as a safe method. Among most threatening complications are hemorrhage and peritoneal perforation. We report on two patients who demonstrated intra-abdominal free air on an erect chest X-ray after TEM procedure without other findings of a pneumoperitoneum. We hypothesize that due to the combination of elevated pressures in the retroperitoneal cavity and decreased integrity of the retroperitoneal barrier, insufflated CO_2_ gas can diffuse into the intraperitoneal cavity. Conservative treatment should be considered in patients with free intra-abdominal air postoperatively. However, there should be no suspicion of peritoneal entry during the procedure and the patient should be in generally good condition without severe abdominal symptoms.

## 1. Introduction

Cancer of the rectum is the fifth most common form of cancer in adults worldwide.

Colorectal cancer is preceded by the development of adenomas. Adenomas are common neoplasms of the colorectal tract. Tubulovillous and villous adenomas account for a high incidence of transformation into invasive colorectal cancer [1].

Since its introduction in 1983, TEM has emerged as a safe and effective method to treat a variety of rectal lesions including adenomas, early stage carcinomas, and strictures [2]. Among most frequent perioperative complications are hemorrhage and peritoneal perforation. Intra-abdominal air without peritoneal perforation is extremely rare. We report on two patients who showed intra-abdominal free air on an erect chest X-ray after TEM procedure. Relaparoscopy in one patient and close followup in the other patient revealed no signs of peritoneal entry, and after conservative treatment the patients were discharged in good health. 

## 2. Case Report

### 2.1. Patient A

A 66-year-old man, previously diagnosed with a papillary thyroid carcinoma and pulmonary metastasis for which he was treated by total thyroidectomy, cervical lymph node dissection, and thoracotomy, was referred to our hospital with changed bowel habits. Digital rectal examination and rectoscopy revealed a tumour at 5 cm from the anal verge. Endoscopic ultrasonography disclosed extension into the rectal wall.

**Figure 1:**
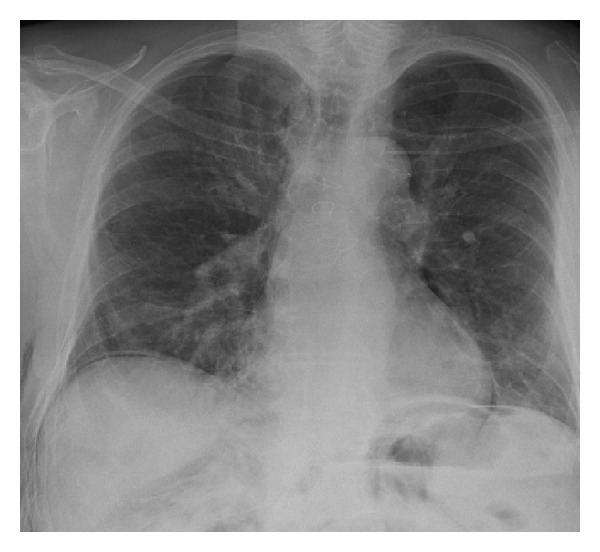
Intra-abdominal free air on an erect chest X-ray.

**Figure 2:**
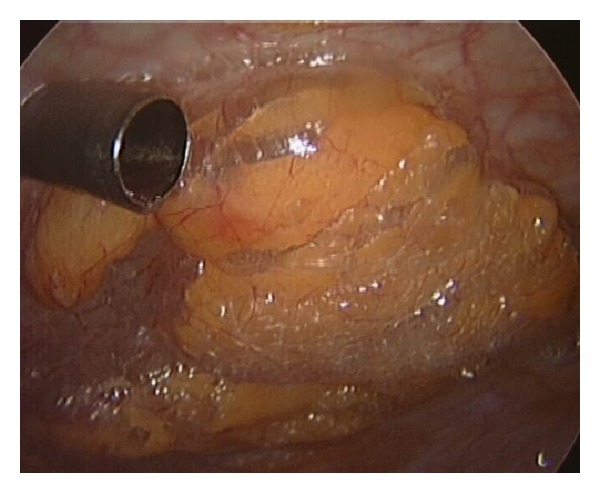
Bulging peritoneum during relaparoscopy without a peritoneal defect.

A TEM procedure was successfully performed in our clinic. Insufflation rate was 6 L CO_2_/minute and intraluminal pressure was 15 mmHg. No signs of peritoneal perforation were observed during the procedure. Histological examination confirmed the diagnosis of a tubulovillous adenoma with a maximum diameter of 5.5 cm. One day after the procedure the patient was in mild discomfort with a body temperature of 39 degrees Celsius. Blood pressure and pulse rate were normal. The abdomen was slightly painful on palpation in the left lower quadrant without muscle guarding. Crepitations on palpation were identified, suggesting the existence of subcutaneous emphysema. Blood analysis showed a leukocytosis (18,000/mm^3^) and elevated c-reactive protein levels (148 mg/L). Conservative treatment was started by broad spectrum antibiotics i.v. (ceftriaxone 1dd 2 gr, metronidazol 3dd 500 mg). An erect X-ray of the chest was performed which showed free air in the abdomen and subcutaneous emphysema (Figure 1). Under suspicion of peritoneal perforation a diagnostic laparoscopy was performed. However, during the procedure no peritoneal defect could be identified. A bulging peritoneum was seen (Figure 2). Conservative treatment was continued and the patient was closely monitored with repeated physical examinations and blood analyses. The patient recovered clinically and blood analyses normalised. Four days after the diagnostic laparoscopy medication was switched to oral antibiotics and he was discharged in good health. Subcutaneous emphysema was present for over 10 days. At one month followup, the patient is initially suffering from rectal cramps but is free of abdominal complaints at the next visit. 

### 2.2. Patient B

A 65-year-old man with hypertension and a rectal sessile polyp in his past medical history was referred to our department. The patient suffered from rectal bleeding. During digital rectal examination a weak mass could be palpated in the anterior wall of the rectum at 3 cm of the anal verge. Rectoscopy was consistent with physical examination. Endoscopic ultrasonography was performed disclosing extension into the rectal wall. Magnetic resonance (MR) imaging scan showed a distal rectal mass with measurements of 3.5 × 2.0 cm without increased lymph nodes. Biopsies raised the suspicion of adenocarcinoma. 

The patient underwent a TEM procedure and the tumour was successfully removed. Insufflation rate was 6 L CO_2_/minute and intra-luminal pressure was 15 mmHg. During the procedure no peritoneal entry was observed. Pathological examination confirmed the diagnosis of T2 adenocarcinoma, radically removed. Close observation of the patient revealed a temperature of 39 degrees Celsius one day after the procedure. On physical examination, the lower abdomen was slight painful without muscle guarding. In his left flank crepitations were identified on palpation, suggesting the existence of subcutaneous emphysema. Blood analysis revealed a leukocytosis (20,500/mm^3^) and an elevated c-reactive protein level (158 mg/L). An erect chest X-ray showed both intra-abdominal free air and subcutaneous emphysema. Intravenous broad-spectrum antibiotics were started (ceftriaxone 1dd 2 gr, metronidazol 3dd 500 mg). After an initial rise in c-reactive protein with a maximum of 261 mg/L, laboratory findings normalised within 4 days. At day 4 the fever resolved, and abdominal examination normalised. The patient was discharged the same day in good health. The patient was given adjuvant treatment by chemoradiation. 

## 3. Discussion

Due to its minimally invasiveness and its local surgical radicality TEM has become the preferred method in the past decades for the curative treatment of rectal neoplasms. Compared to major rectal surgery, morbidity and mortality are extremely low [3–6]. Complication rates vary in literature ranging from 6%–31%. Most common major complications include peritoneal entry and postoperative hemorrhage with a complication rate of 0%–9% and 1%–13%, respectively [7].

It has been shown that peritoneal perforation not necessarily demands conversion to laparotomy [8]. Instead, it can be managed by primary closure of the defect without increased major or minor complication rates postoperatively. Furthermore, no difference in hospital length of stay was observed between patients with peritoneal entry to those without [6].

Postoperatively, suture line dehiscence could occur after primary closure of peritoneal perforations creating an open connection between the rectum and the intraperitoneal cavity. Those patients are prone for intra-abdominal infections and most likely suffer from abdominal pain and fever. In our first patient, we were confronted with fever and free air on the erect chest X-ray. On diagnostic laparoscopy, we failed to find defects in the integrity of the peritoneum. However, a bulging impression of the peritoneum suggested increased retroperitoneal pressures (Figure 2). Laparoscopy was aborted in favor of conservative treatment. Since the postoperative presentation of our second patient was similar to the first, we successfully decided to start conservative treatment with close followup. Fever after TEM procedures, known as “TEM-fever”, is a common condition as the vast majority shows fever postoperatively. This was concluded by oral communication during the TEM meeting in Bergen, 2011. To our knowledge, intra-abdominal free air without a defect in the peritoneum has never been described to date. Increased rectal pressure during the TEM procedure moves insufflated CO_2_ gas via loose connective tissue to the retroperitoneal cavity resulting in subcutaneous emphysema. We hypothesize that due to the combination of elevated pressures in the retroperitoneal cavity and decreased integrity of the retroperitoneal barrier, insufflated CO_2_ gas diffused into the intra-peritoneal cavity. We present two cases with free abdominal air after TEM procedure without peritoneal perforation. Therefore, we state that conservative treatment should be considered in those patients. However, there should be no suspicion of peritoneal entry during the procedure and patients should be in generally good condition with no septic signs or abdominal symptoms. Close monitoring of the patient is necessary, so that conservative management can be replaced by surgery the moment the patient deteriorates.
